# TP53: an oncogene in disguise

**DOI:** 10.1038/cdd.2015.53

**Published:** 2015-05-29

**Authors:** T Soussi, K G Wiman

**Affiliations:** 1 Department of Oncology-Pathology, Karolinska Institutet, Cancer Center Karolinska (CCK) R8:04, Stockholm SE-171 76, Sweden; 2 Sorbonne Universités, UPMC Univ Paris 06, Paris F-75005, France; 3 INSERM, U1138, Centre de Recherche des Cordeliers, Paris, France; 4 Université Paris Descartes, Sorbonne Paris Cité, Paris, France

## Abstract

The standard classification used to define the various cancer genes confines tumor protein p53 (TP53) to the role of a tumor suppressor gene. However, it is now an indisputable fact that many p53 mutants act as oncogenic proteins. This statement is based on multiple arguments including the mutation signature of the TP53 gene in human cancer, the various gains-of-function (GOFs) of the different p53 mutants and the heterogeneous phenotypes developed by knock-in mouse strains modeling several human TP53 mutations. In this review, we will shatter the classical and traditional image of tumor protein p53 (TP53) as a tumor suppressor gene by emphasizing its multiple oncogenic properties that make it a potential therapeutic target that should not be underestimated. Analysis of the data generated by the various cancer genome projects highlights the high frequency of TP53 mutations and reveals that several p53 hotspot mutants are the most common oncoprotein variants expressed in several types of tumors. The use of Muller's classical definition of mutations based on quantitative and qualitative consequences on the protein product, such as ‘amorph', ‘hypomorph', ‘hypermorph' ‘neomorph' or ‘antimorph', allows a more meaningful assessment of the consequences of cancer gene modifications, their potential clinical significance, and clearly demonstrates that the TP53 gene is an atypical cancer gene.

## Facts

- p53 mutants are among the most common protein variants expressed in cancer cells.
- Classifying TP53 status in human cancer as ‘inactivated' or ‘loss-of-function' is misleading.
- Many mutant p53 variants are oncogenic with multiple GOF activities essential for neoplastic transformation.

## Open Questions

- How does the diversity of oncogenic p53 variants contribute to the heterogeneity of the malignant phenotype?
- What is the contribution of p53 protein accumulation in human tumors to the GOF of mutant p53?
- Is there a tissue specificity of mutant p53 GOF?
- What will be the best strategy to target oncogenic p53 mutants for improved cancer therapy?
- Should the binary classification oncogene—tumor suppressor gene be replaced with a functional classification based on the activity of the mutated variant?

The development of next-generation DNA sequencing methods has led to a burst of information, including the release of the sequences of >5000 cancer genomes, an achievement inconceivable only 15 years ago.^1, 2^ With the aim of understanding tumor development and finding novel genetic biomarkers to improve patient care or define new therapeutic targets, these analyses have led to the creation of lists describing the ‘most significant mutated genes' in various types and subtypes of cancer.^3, 4, 5^ Well known cancer genes such as TP53, PIK3CA (phosphoinositide-3-kinase, catalytic, alpha polypeptide), adenomatous polyposis coli gene (APC) and KRAS (v-Ki-ras2 Kirsten rat sarcoma 2 viral oncogene homolog) are still at the top of these lists, but novel genes with potential clinical value have also been identified. However, their relatively infrequent mutation and/or association with specific tumor groups could limit their value as biomarkers or therapeutic targets.^6^

In general, cancer genome studies highlight oncogenes as the most promising targets for drug development mainly because these hyperactive protein variants appear to be more easily druggable than the products of tumor suppressor genes, inactivated by heterogeneous nonsense or frameshift mutations.^7^ Therefore, drug discovery and development programs in academia and industry have mostly focused on kinase inhibitors and other approaches designed to inhibit activated oncoproteins, whereas relatively few therapeutic strategies to reactivate tumor suppressors have been developed to date.

The first evidence for the existence of tumor suppressor genes came from cell fusion experiments performed by Harris *et al.*^8^ in the 1960s, showing that fusion of normal cells with tumor cells resulted in a normal phenotype. Loss of specific chromosomes from the hybrid cells led to reappearance of the tumor phenotype, suggesting the existence of critical genes able to suppress the tumor phenotype. The tumor suppressor gene concept was also supported by Knudson's epidemiological studies on familial and sporadic retinoblastoma and the subsequent loss of heterozygosity (LOH) analysis that identified chromosomal deletions in tumors.^9, 10^ Haber and Harlow defined tumor suppressor genes as ‘genes that sustain loss-of-function (LOF) mutations in the development of cancer'.^11^ This classification has evolved over time and novel classes such as caretakers, gatekeepers or landscapers have been added to take into account the function of novel genes and their association with tumor development.^4, 12^ Although oncogene and tumor suppressor gene alterations may have direct consequences on cell growth, caretakers and landscapers act indirectly by promoting either genetic instability or an abnormal cellular environment that will foster neoplastic transformation. This classification, based on the diversity of the functional activity of the wild-type product, should not be confused with the consequences of their alteration.

For example, LOF is still the main characteristic of tumor suppressor genes, but the Haber and Harlow definition does not take into account the heterogeneity of the different protein variants or other activities such as dominant negative (DN) effects on wild-type protein or gain-of-function (GOF). Furthermore, for some genes such as Notch, this definition can be cell-type specific, as the spectrum of mutations is different in blood and solid tumors, with different consequences for the protein.^13, 14^ Although the two-box oncogene/tumor suppressor categorization remains a central concept in tumor biology, it is becoming increasingly obvious that it is difficult to put some genes into one box. As cancer research develops rapidly, it is essential to ensure highly flexible data classification so that labels do not paralyze the emergence of new concepts.

The status of TP53 as a tumor suppressor gene can be used as a paradigm. Wild-type TP53 clearly acts as a negative regulator of cell growth, but considering TP53 mutations solely as LOF mutations would prevent a full understanding of how TP53 mutations drive tumor growth, as TP53 status in human cancer is often defined in binary terms, wild-type *versus* inactivated, despite accumulating evidence that the majority of mutant p53 proteins are heterogeneous oncogenic proteins with multiple GOF activities and with potential as therapeutic targets.

In this review, we will show that, despite the important diversity of TP53 mutations, some specific p53 mutants are among the most frequent variants expressed in human cancer. In addition, we have gathered various lines of evidence accumulated over 35 years of research that illustrate how TP53 is an out-of-the-box entity. Finally, using Muller's classical definition of mutations based on quantitative and qualitative consequences on the protein product, we will discuss how this classification would be more appropriate to define the heterogeneous effects of cancer gene mutations.

## Pattern of TP53 mutations in human cancer: oncogenic hotspot mutant TP53 is one of the most frequently expressed protein variants in human cancer

**Figure 1:**
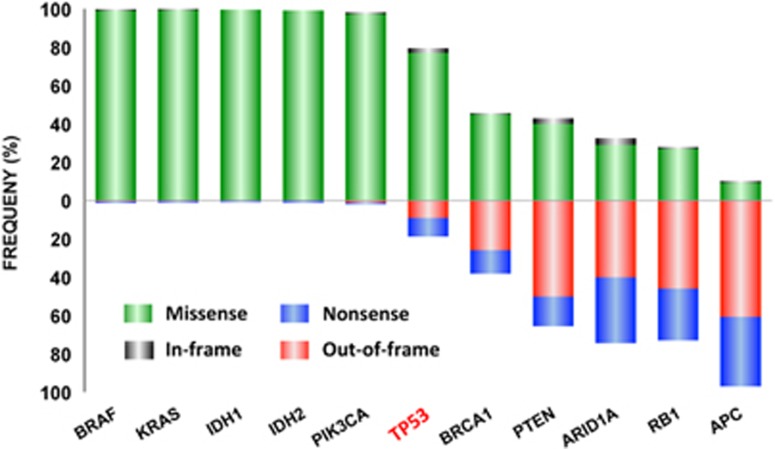
Mutation spectrum of frequently mutated genes in human cancer—Green: missense mutations; Grey: in-frame insertions and deletions; red: out-of-frame insertions/deletions and splice mutations; orange: nonsense mutations; Blue: nonsense mutations. Data were obtained from the cosmic database (http://cancer.sanger.ac.uk/cancergenome/projects/cosmic/) except for those of the TP53 gene, obtained from http.p53.fr. BRAF: v-raf murine sarcoma viral oncogene homolog B1: KRAS, v-Ki-ras2 Kirsten rat sarcoma 2 viral oncogene homolog; IDH1, isocitrate dehydrogenase 1; IDH2, isocitrate dehydrogenase 2; PIK3CA, phosphoinositide-3-kinase, catalytic, alpha polypeptide; TP53, tumor protein p53; BRCA1, familial breast/ovarian cancer gene 1; PTEN, phosphatase and tensin homolog gene; ARID1A, AT-rich interactive domain 1 A; RB1, retinoblastoma gene; APC, adenomatous polyposis coli gene

A unique feature of the TP53 gene compared with other tumor suppressor genes is its mode of inactivation.^15, 16, 17, 18^ More than 80% of somatic and germline TP53 alterations are missense mutations that lead to the synthesis of a stable mutant protein that accumulates in the nucleus of tumor cells (Figure 1). This high frequency of substitutions is highly analogous between the various types of cancer despite a different spectrum of mutation due to variable exposure to carcinogens.

Oncogenes are typically activated by missense mutations that target specific key residues of the protein (Figure 1). Tumor suppressor genes display either out-of-frame insertions and deletions (indel) or nonsense mutations, both leading to loss of protein expression (Figure 1). These patterns of cancer-associated mutations have been used to classify cancer genes as either oncogenes or tumor suppressor genes in the absence of data on the functional consequences of the mutations. In a recent review on cancer genomics, Volgelstein *et al.*^4^ proposed a ratiometric method to classify cancer genes based on the frequency of recurrent mutations and type of alterations (missense *versus* nonsense or frameshift). Although their method can separate classical oncogenes from tumor suppressor genes in general, the TP53 gene would still straddle the boundary between the two classes of genes.

The strong selection to maintain expression of full-length p53 protein in tumors is highly suggestive of a vital role in transformation, including DN activity and/or GOF. As >90% of TP53 mutations are localized in the core domain of the protein (residue 100 to 300), all p53 protein isoforms are affected by these alterations.^19^

The distribution of mutations in the p53 protein is also unique among all cancer genes, including oncogenes and tumor suppressor genes. All but 7 residues of the 393 amino acid residue p53 protein have been the target of at least 1 mutation in human cancer, and in the core domain that contains the DNA-binding region, each residue has been found to be mutated at least 5 times in independent tumors, and up to >2,000 times for hotspot mutants.^19, 20^ This vast scattering of TP53 mutations is due to the marked fragility of the core domain, which can be destabilized by amino acid substitutions at many different positions.^21^

**Figure 2:**
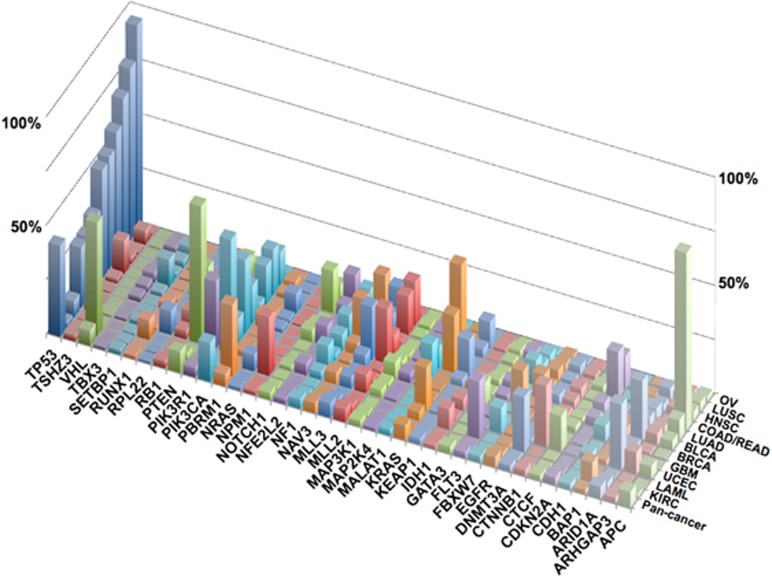
Genes most frequently mutated in various types of cancer in the Pan-Cancer study. Only the 40 most significantly mutated genes in the Pan-Cancer study are shown on this graph. The PAN-CANCER study included glioblastoma multiforme (GBM), lymphoblastic acute myeloid leukemia (LAML), head and neck squamous carcinoma (HNSC), lung adenocarcinoma (LUAD), lung squamous carcinoma (LUSC), breast carcinoma (BRCA), kidney renal clear-cell carcinoma (KIRC), ovarian carcinoma (OV), bladder carcinoma (BLCA), colon adenocarcinoma (COAD), uterine cervical and endometrial carcinoma (UCEC) and rectal adenocarcinoma (COADREAD). Pan-cancer: integrated data with all cancer types. Data were generated by analysis of the mutations released by Kandoth *et al.*^3^

**Figure 3:**
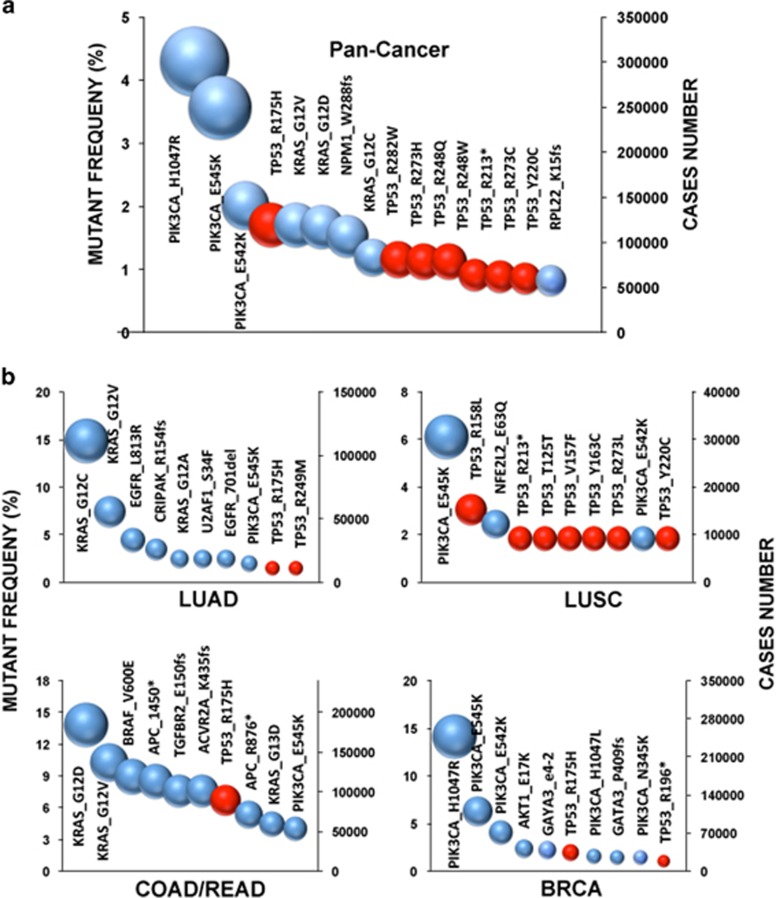
Most frequent protein variants in human cancer. (**a**) Fifteen most frequent protein mutants in the 12 types of cancer included in the Pan-Cancer study. (**b**) Ten most frequent protein mutants in four different types of cancer. Left *y*-axis: mutant variant frequency; Right *y*-axis: number of cancer worldwide associated with the different variants. p53 mutants are shown in red. Hotspot p53 mutants found in LUSC, such as p.R158L or p.V157F, are specific hotspot mutations associated with smoking. Frequencies for other types of cancer are shown in Supplementary Figure 1. This analysis included lung adenocarcinoma (LUAD), lung squamous carcinoma (LUSC), breast carcinoma (BRCA) and colon and rectal adenocarcinoma (COAD/READ)

The Cancer Genome Atlas Pan-Cancer project has released an integrated set of genomic data from 3,200 cancer patients with 12 tumor types.^3, 22^ The data include genomic, epigenomic, transcriptional and proteomic information. Not surprisingly, TP53 was found to be the most frequently mutated gene in this new set of data (Figure 2 and Supplementary Figure 1). This leading position for TP53 was already obvious before the various cancer genome sequencing projects were launched and it remains unchallenged today, as most novel cancer genes are either cancer-specific (IDH1 and IDH2), mutated at low frequency (FBXW7 or GATA3) or both. One of the drawbacks of using a classification based on gene mutation frequency is that it does not take into account the diversity of the different protein variants for each gene. Although KRAS mutations are restricted to a few codons, those in other genes may be scattered along the coding region, which leads to multiple protein variants and hinders potent information on mutant diversity. Using mutational data released by the Pan-Cancer project, we have been able to perform an integrated analysis focusing directly on each protein variant (Figure 3). Three PIK3CA variants (p.H1047R, p.E545K and p.E542K) were the most frequent mutants found in the 12 tumor types; they were found in 9.9% of patients, corresponding to ~300 000 cancer cases worldwide. This observation clearly supports the importance of this gene as a target for therapy.^23^ Mutant p53 p.R175H ranked fourth in this analysis and 6 other p53 hotspot mutants were among the 15 most frequent protein variants found in human cancer (Figure 3a). The inclusion of other common cancers such as gastric cancer or hepatocellular carcinoma with a high frequency of TP53 mutation would provide a more accurate estimate of the number of cancer cases associated with specific p53 variants. When specific cancer types are considered, p53 mutants are always among the 10 most frequent protein variants, except in kidney cancer and acute myeloid leukemia (AML; Figure 3 and Supplementary Figures 1A–G). In ovarian cancer, the high frequency of TP53 mutation (>90%) and the lack of other frequently mutated genes leads to a distribution of protein variants that includes only p53 mutants (Supplementary Figures 1A–G). This analysis clearly shows that mutant p53 are among the most frequent protein variants expressed in human cancer and, as discussed below, these individual oncogenic proteins should not be ignored as potential targets for cancer therapy. Apart from their high frequency, these mutations are also highly ubiquitous, associated with various tumor types, and, in many cases, correlated with aggressive tumor phenotypes and poor prognosis.^24, 25^

## Mutant p53 heterogeneity: LOF and GOF

Before addressing the various GOF activities of mutant p53, it is essential to discuss the reasons for one of the most striking features of mutant p53, namely p53 protein accumulation in tumor cells. This topic was overlooked for a long time, perhaps due to the widespread belief that this feature was inherent to the mutant protein which displayed an increased half-life. More recently, this issue has been revisited, as targeting mutant p53 accumulation could be a very promising approach for the development of therapy.

The increased levels of nuclear p53 in tumor cells were described more than 3 decades ago but the relation with TP53 mutation became clear 10 years later and immunohistochemistry analysis of tumors using various p53 monoclonal antibodies was developed as a surrogate for p53 variant analysis.^26, 27^ Several observations argued that p53 protein accumulation was not an intrinsic property of the TP53 protein. Approximately 70% of individuals with the Li-Fraumeni syndrome carry a heterozygous germline mutation in the TP53 gene and express both wild-type and mutant p53 in their normal cells. Despite this genotype, accumulation of mutant p53 protein can only be visualized in tumors, whether or not the wild-type p53 allele is retained.

Terzian *et al.*^28^ used a knock-in mouse model to analyze the expression of mutant p53 in various tissues. Their first observation was that mouse tumors behaved like human tumors; they displayed mutant p53 accumulation in the nucleus, whereas mutant p53 was undetectable in normal cells. This feature was observed in both heterozygous (one mutant and one wild-type allele) and homozygous (two mutant alleles) animals. This is an important finding because it rules out the possibility that the inability of mutant p53 to act as a transcription factor and induce sufficient levels of MDM2 protein, a p53-specific E3 ubiquitin ligase that targets p53 for modification and subsequent degradation by the 26 S proteasome, is a cause for mutant p53 hyperstability.

However, additional ubiquitin ligases have recently been identified that participate in the degradation of the tumor suppressor, including Pirh2 (p53-induced protein with a RING-H2 domain), Trim24 (transcription intermediary factor 1-alpha), COP1 (constitutive photomorphogenesis protein 1) and CHIP (C-terminus of Hsc70 interacting protein).^29, 30^ The CHIP E3 ligase participates in p53 degradation by forming a large complex that includes HSP90 and HSP70. Mutant p53 forms stable complexes with both HSP, CHIP and MDM2, inhibiting the ligase activity of both. A large survey of tumor cell lines expressing wild-type and mutant p53 showed that stabilization of endogenous mutant protein is due to a complete lack of ubiquitination.^31^ Inhibition of HSP90, either via knockdown or by specific drugs, alleviates the formation of such complexes, leading to reactivation of both MDM2 and CHIP, and mutant p53 degradation.

The heterogeneity of p53 mutants was discovered 25 years ago with the description of the so-called ‘structural' mutants, in which the DNA-binding domain is unfolded (e.g. p.R175H), and ‘DNA contact' mutants, in which residues interacting directly with DNA are substituted (e.g., p.R273H).^32, 33, 34, 35^ Nuclear magnetic resonance, circular dichroism and X-ray diffraction analyses have confirmed and expanded this description and suggested that multiple thermodynamic stages are associated with the various mutations depending on their position.^36, 37, 38^ This heterogeneity extends to the biological activity of mutant p53 proteins.^39^ Analysis of the transcriptional activity of 2500 p53 mutants on 8 different target genes representative of different TP53 functions demonstrated heterogeneous penetrance of the various p53 mutants with >50% presenting only partial loss of activity.^40, 41, 42, 43^

Mutant p53 GOF has been extensively investigated in multiple *in vitro* and *in vivo* systems (see refs 18 and 44, 45, 46, 47 for reviews). GOF was hypothesized as early as 1993, when Dittmer et al. showed that the introduction of different p53 mutants into TP53-null cells resulted in an oncogenic phenotype such as enhanced tumorigenic potential^48^ (It is also possible to date GOF several years earlier, when mutant TP53 misidentified as wt TP53 was shown to transform cells. Whether or not reinterpretation of these results in the light of new data can predate subsequent observations is an endless semantic debate^44^). Further studies have largely supported this observation. Mutant p53 GOF includes enhanced tumorigenesis, metastasis, resistance to therapy and genomic instability. The genomic instability effect was further supported in a recent analysis of 3000 tumors showing that TP53 mutations were strongly associated with a high frequency of copy number changes.^49^ GOF in p53 mutants is highly heterogeneous, an observation that may be related to the various conformations of p53 mutants. GOF mechanisms may be the result of changes in the specificity of the DNA-binding activity of the p53 mutant, leading to the induction of novel transcriptional programs, or changes of its interaction with other cellular proteins directly or indirectly related to the regulation of gene expression. Several transcriptional programs are specifically activated by p53 mutants, most of them resulting in increase of tumorigenicity but whether this is mediated by a direct binding of the mutated protein to a specific DNA sequence or via an interaction with other transcription factors remains to be elucidated^50, 51, 52^ (see ref. 53 for review). A recent study from Myers and co-workers^54^ shows that several p53 mutants exert a transcription-independent GOF by downregulating the AMPK (AMP-activated protein kinase), an observation adding one more grain to the important relation between TP53 and metabolism. This observation is also in line with the study by Zhang *et al.*^55^ showing that p53 mutants stimulates the Warburg effect in cancer cells.

The discovery that the p53 family consists of three members (TP53, TP63 and TP73) increased the complexity of this network, as the two p53 homologs might also contribute to its oncogenic potential.^56, 57^ TP63 and TP73 are both expressed as many isoforms due to alternative usage of promoters for transcription and alternative splicing. Long isoforms (p73 or p63 containing the transactivation domain (TA-p73 or TA-p63)) are able to transactivate the same target genes as p53 and induce apoptosis, while short forms (amino-deleted p63 or p73 isoforms (DN-p63 or DN-73)) have an opposite activity via DN mechanisms. TP63 and TP73 are able to cooperate with TP53 to induce apoptosis, suggesting the existence of a complex network of interactions between the products of these three genes. p53 mutants with unfolded structure, but not DNA contact mutants, bind specifically to p63 and p73 and impair their apoptotic activity (see refs 58, 59, 6061 for review).

An important issue that has never been fully analyzed is the consequence of accumulation of mutant p53 protein in tumor cell nuclei. Studies have shown that this accumulation may be as much as 10-100-fold higher than that of wild-type protein, which raises questions concerning the specificity of some mutant p53 activities and how some nonspecific squelching effects may be associated or confounded with these novel properties. Furthermore, this aspect also raises the issue of tumor heterogeneity, as p53 accumulation in human tumors is highly heterogeneous, even for single p53 variants, and could be associated with the genetic background of the tumor and the individual.

The general idea that loss of transcription in mutant p53 is the driving force selected during tumorigenesis also needs to be reevaluated, as it is far from straightforward. Surprisingly, mice expressing p53 mutants transcriptionally defective for the three canonical pathways, growth arrest, senescence and apoptosis, are not prone to cancer.^62, 63^ Cells from mice deficient for the three major TP53 target genes, CDKN1A (the gene encoding p21), BBC3 (the gene encoding PUMA) and PMAIP1 (the gene encoding Noxa) (p21−/−puma−/−noxa−/− mice) are deficient in their ability to undergo p53-mediated apoptosis, G1/S cell-cycle arrest, and senescence, but these animals also remain tumor-free.^64^ Taken together, these observations suggest that TP53 driver mutations are selected to impair specific TP53 pathways that remain to be identified, and only mutants defective in such transcriptional programs will be selected in human tumors.^65^

### Mouse models

The first TP53 knockout mice were generated by removing exons encoding the DNA-binding domain, thus impairing the expression of all p53 isoforms.^66^ Homozygous TP53−/− mice are highly prone to cancer, in particular T-cell lymphoma and sarcoma. Although these knockout mice supported the model of TP53 as a tumor suppressor gene, they were not fully satisfactory, since most cancers in humans are carcinomas, which were observed at very low frequency in TP53 knockout mice.^67, 68^ Improvements in the production of transgenic mice led to the creation of knock-in mice expressing various hotspot mutants that include structural or DNA contact mutants and result in more ‘human-like' tumors.^69, 70^ Compared with TP53−/− mice, these novel knock-in models displayed a higher degree of heterogeneity in the spectrum of tumor types with more frequent carcinomas. Furthermore, these tumors were highly invasive and metastatic, a feature absent in TP53−/− mice. Two characteristics of these mice models support the notion of heterogeneous GOF for p53 mutants. First, the spectrum of tumors differed according to the various mutant alleles used in these mice. For example, the ‘DNA contact' mutant p.R270H (p.R273H in humans) results in a high frequency of carcinomas, whereas the ‘structural' mutant p.R172H (p.R175H in humans) predominantly results in osteosarcomas. Second, the tumor spectra of heterozygous TP53 p.R172H/− or TP53 p.R270H/− mice were different from those of TP53+/− or TP53−/− mice, which argues against any differences due to a DN activity toward wild-type p53. Some p53 mutants such as p.R175P are defective in activating genes associated with apoptosis without impairing growth arrest.^71^ Onset of tumors in knock-in mice expressing this mutant (p.R172P in mice) is delayed compared with TP53−/− mice or mice expressing hotspot mutations.^72^ In contrast to tumors expressing other p53 mutants, these tumors were mostly diploid, indicating that the growth arrest function of TP53 could be essential for maintaining genetic stability, but is not the primary tumor suppressor function of wild-type TP53.

### Targeting mutant p53 for novel cancer therapy

**Figure 4:**
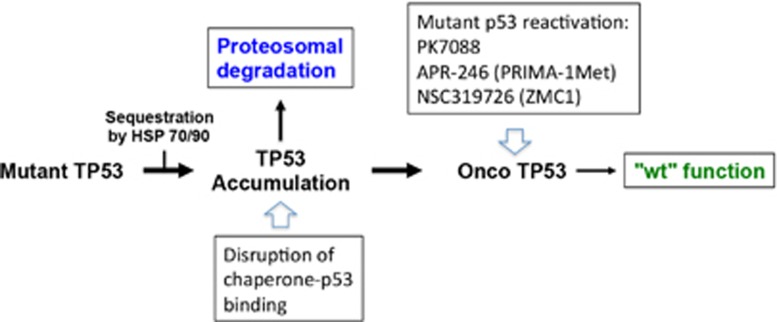
Strategies for therapeutic targeting of mutant TP53. 1: p53 turnover is dependent on E3 ligases such as MDM2 or CHIP that mediate ubiquitination and degradation of TP53. In tumor cells, TP53 protein as well as MDM2 and CHIP can be sequestered in high molecular weight complexes by chaperone proteins HSP70 and HSP90. This blocks the ubiquitin ligase activity of MDM2 and CHIP, leading to mutant TP53 accumulation which is fundamental for its GOF activities. Such accumulation can be targeted by drugs that disrupt TP53-chaperone binding, allowing proteosomal degradation of TP53. Furthermore, small molecules such as PK7088, APR-246 and NSC319726 have been shown to target specific or multiple forms of mutant TP53—Onco TP53—and promote TP53 refolding. This leads to restoration of TP53-dependent transcription and reactivation of TP53 biological responses such as cell-cycle arrest, senescence and apoptosis. It may also inhibit pathways associated with TP53 GOF, including binding and inactivation of TP53 family member proteins TP63 and TP73 ^74, 100^

Many investigators have initiated efforts to develop novel strategies for pharmacological reactivation of mutant p53 in cancer cells.^73, 74^ This is a major challenge, for several reasons. Mutant p53 is clearly a different kind of target compared with those of successful novel anticancer agents such as Herceptin (trastuzumab) and Gleevec (imatinib) that block critical oncogenic kinases overexpressed in various tumors. In the case of mutant p53, the main aim is to refold and reactivate a dysfunctional tumor suppressor (Figure 4). Moreover, the protein target in this case is a DNA-binding transcription factor, a type of protein target that has been considered not easily ‘druggable'. To make things more complicated, the structural heterogeneity of mutant p53 proteins raises concerns as to the feasibility of designing or identifying therapeutic agents that can rescue more than one specific mutant form of p53 or a subset of mutants. However, the realization that mutant p53 can have GOF activity may open possibilities for therapeutic strategies designed to inhibit these functions, which may be easier to achieve than full restoration of wild-type function.

Researchers have approached the challenge of mutant p53 reactivation in various ways (Figure 4). Fersht and co-workers^75^ used detailed structural information from NMR and/or X-ray crystallography as the starting point for rational design of small molecules that can bind and stabilize the wild-type conformation of the p.Y220C hotspot p53 mutant, which occurs in ~75 000 cases of cancer per year worldwide. Structural studies have shown that the substitution of cysteine for tyrosine at position 220 gives rise to a destabilizing crevice in the p53 protein. Fersht *et al.*^76^ have designed compounds such as the carbazole derivative PK083 that bind to the crevice and raise the melting temperature of the mutant protein.

The compound PK7088 also raises the melting temperature of the p.Y220C mutant, and triggers cell-cycle arrest and apoptosis in tumor cells in a p.Y220C-dependent manner.^77^ PK7088 increases the fraction of correctly folded p.Y220C mutant p53 in cells and enhances expression of the TP53 targets p21 and Noxa. Synergy with the MDM2-inhibiting compound Nutlin-3 was also observed, supporting the proposed mechanism of action.

NSC319726 (ZMC1) is another mutant-specific p53 reactivator. Carpizo and co-workers^78^ identified this thiosemicarbazone compound based on analysis of the NCI database and showed that it specifically targets the p53 hotspot mutant p.R175H. NSC319726 can restore wild-type conformation and function to this mutant and trigger p.R175H-dependent cell death by apoptosis.^78^ Moreover, NSC319726 suppresses growth of tumor xenografts expressing the p.R175H mutant in mice. NSC319726 has zinc ion-chelating properties that seem to be important for its ability to reactivate p.R175H mutant p53. A subsequent study provided further data on the zinc-metallochaperone activity of NSC319726, and showed that the compound can rescue several other p53 mutants with impaired zinc binding, for example, p.G245S.^79^ This study also highlighted the ability of NSC319726 to induce reactive oxygen species (ROS) in cells.

The small molecules PRIMA-1 (p53 reactivation and induction of massive apoptosis-1) and MIRA-1 (mutant p53-dependent induction of rapid apoptosis-1) were identified in a cell-based screen of the NCI Diversity set.^80, 81^ PRIMA-1 and its structural analog PRIMA-1Met (APR-246) refold mutant p53, enhance expression of several TP53 targets, including Bax, Puma and Noxa, and inhibit human tumor xenografts in SCID mice and mouse tumors in syngeneic hosts. These effects are observed with a range of mutant p53 proteins. APR-246 synergizes with several chemotherapeutic drugs, for example, adriamycin and cisplatin, to induce mutant p53-dependent tumor cell death.^82^ Both PRIMA-1 and APR-246 are converted to the Michael acceptor methylene quinuclidinone (MQ) that binds covalently to cysteines in the p53 core domain^83^ MQ binding to p53 is sufficient for mutant p53 reactivation, as shown by protein transfer experiments with APR-246/MQ-treated recombinant p53. It is currently not clear to which p53 cysteines MQ binds, although docking simulations and functional studies in cells have indicated that C124 is one possible target.^84^ APR-246 has been tested in a phase I/II clinical study in patients with hematological malignancies or hormone-refractory prostate cancer.^85^ This study showed that APR-246 is safe and has a favorable pharmacokinetic profile. Biological effects consistent with p53 reactivation were observed in patient leukemic cells after treatment, and clinical responses were observed in two patients, including one AML patient with a p53 p.V173M core domain mutation.

In addition to targeting p53, APR-246 has been shown to inhibit thioredoxin reductase (TrxR1) and convert the enzyme to an NADPH oxidase,^86^ thereby increasing cellular ROS levels. Moreover, Tessoulin *et al.*^87^ found that APR-246 can decrease GSH levels and thus impair the redox balance in multiple myeloma cells independently of p53.

Various other small molecule reactivators of mutant p53 have also been identified, including CP31398, Ellipticine, P53R2, SCH529074 and stictic acid reviewed in the study by Bykov and Wiman.^74^ Their mechanisms of action are not fully understood and they have not yet been tested in the clinic.

In light of the growing evidence in favor of GOF activities of mutant p53, a more modest but still attractive aim for therapeutic targeting of mutant p53 is inhibition of the oncogenic properties of mutant p53 (Figure 4). This may be easier to achieve than full restoration of wild-type function. One such approach consists of disruption of mutant p53 binding to TP63/p73. This has been shown for the small molecule RETRA (reactivation of transcriptional reporter activity).^88^ Disruption of complexes between mutant TP53 and TP63/p73 by RETRA leads to restoration of expression of TP53 target genes and tumor suppression.

A significant fraction (8%) of TP53 mutations are nonsense mutations that give rise to expression of a truncated and inactive p53 protein. The c.637C>T (p.R213*) mutant is the most common nonsense mutation in TP53, and is actually more common than many missense mutations in human tumors^19^ (see also http://p53.free.fr). Restoration of nonsense TP53 mutations will obviously require different approaches than those described above for missense mutations. Interestingly, aminoglycosides, for example, gentamicin and G418, have been shown to induce read-through of the c.637C>T (p.R213*) mutant p53 and expression of full-length p53.^89^ Although clinical use of these drugs is limited by their toxicity, the results nonetheless demonstrate that induction of read-through of premature stop codons in nonsense mutant p53 is feasible and suggest that high throughput screening for more efficient and less toxic read-through-inducing compounds should be carried out. Questions remain as to the activity of the full-length p53 protein induced on translational read-through and to what extent pharmacological induction of read-through of premature stop codons will induce read-through of natural stop codons. If these problems can be solved, induction of read-through could be a useful strategy not only for reactivation of nonsense mutant p53, but also for reactivation of other tumor suppressors that are frequently inactivated by nonsense mutations, for example APC and PTEN (phosphatase and tensin homolog gene).

### Cancer gene classification

In Muller^90^ suggested a classification of mutations based on quantitative analysis of wild-type ‘characters'. He proposed the terms ‘amorph, ‘hypomorph', ‘hypermorph', ‘neomorph' or ‘antimorph'. The significance of these terms was subsequently modified, as they were proposed at a time when the relationship between a gene and its product was not clearly established.^90^ These terms can now be used to classify cancer genes to more clearly understand the consequences of cancer gene alterations. This system has enormous benefits, as it is based on the outcome of the mutation regardless of the initial biological function of the gene. It could greatly facilitate the design of optimal drug development strategies.

The term amorph (or LOF) mutation can be applied mainly to tumor suppressor genes, whose function(s) must be totally impaired to drive tumorigenesis, whereas a hypomorph mutation only leads to partial reduction of activity. From a genetic point of view, true amorphic alterations can be easily associated with genes that sustain biallelic deletions such as PTEN or retinoblastoma gene (RB1).^91^ Other mechanisms, such as promoter methylation, loss of expression via microRNA dysregulation or frameshift and nonsense mutations, can also lead to total LOF, but in many cases it is difficult to exclude residual activities that will lead to heterogeneous hypomorphic variants, and for many genes it is likely that true amorphic variants are not as frequent as hypomorphic variants (Table 1). For the APC gene, the size of the truncated proteins has a major influence on several clinical features or genetic events that target the remaining wild-type allele, suggesting that several mutants are hypomorphic.^92^ Dosage reduction via haplodeficiency can also be included among hypomorph mutations. Although many hotspot TP53 mutations are transcriptionally inactive, the majority of the remaining TP53 missense variants are hypomorphic, as they display heterogeneous loss of transcriptional activity.^43^

Antimorphic or DN mutations, as subsequently described by Herskowitz,^93^ have been defined as disruption of the wild-type activity by the mutant polypeptide (Table 1). DN activity was initially defined by hetero-oligomerization of wild-type and mutant protein alleles leading to the formation of an inactive hetero-oligomer, a definition which is restricted to proteins with potential oligomerization activities. The use of the term ‘DN activity' has been largely extended to any type of indirect inactivation of the wild-type allele function or its pathway via the product of the mutant allele. This broad definition can take into account the consequences of multiple mechanisms such as binding and sequestration of cofactors, limiting their availability or the occupancy of transcription promoters by factors that contain inactive transcription domains and therefore act as repressors. Furthermore, DN activity is fairly difficult to assess and distinguish from haplo-insufficiency or GOF. Only mouse models using a combination of knock-in or knockout mutations can resolve these issues. Multiple DN activities have been described for tumor suppressor genes but only for a few genes, such as TP53 or WT1 (Wilm's tumor 1), has it been interpreted in terms of a specific interaction between the wt and mutant allele. The formation of hetero-tetramers between wild-type and mutant p53 has been well documented and many *in vitro* analyses indicate that the activity of poised tetramers, based on transcription assays, is severely impaired. Formation of p53 tetramers obeys a particular kinetic with rapid formation of dimeric molecules followed by slow association of these dimers into tetramers.^94^ Consequently, poised dimers that would contain either a single wild-type or mutant subunit have not been observed and only wt2/Mut2 tetramers are formed. Several mouse models have confirmed the DN activity of certain p53 mutants towards the protein expressed by the wild-type allele, including the hotspot mutant p.R175H.^95^ It remains to be explained why the second TP53 allele, localized on chromosome 17p, is so frequently deleted in human and mouse tumors. Either the DN effect is not complete and full inactivation of TP53 is mandatory for tumor progression, or other genes, localized in the vicinity of TP53, are the true targets of these LOH events. Other tumor suppressor genes such as eukaryotic translation initiation factor 5A (EIF5A) or the potassium channel tetramerization domain containing 11 proteins (KCTD11) are co-deleted with TP53.^96^ Analysis of tumors at this transition from TP53^Mut/wt^ to TP53^Mut/−^ would be highly informative in this regard.

The ‘hypermorph' label is the most explicit term to describe many protein variants expressed from mutated oncogenes, as most of them are hyperactive proteins. For amplified genes such as N-myc, MDM2 or CCND1 (the gene encoding cyclin D1), or genes upregulated via chromosomal translocations such as c-myc, this definition appears to be obvious at first glance, as it refers to the abnormal accumulation of wild-type proteins (Table 1). Nevertheless, it is possible that, at a certain threshold level, the accumulated protein will impair unrelated pathways via nonspecific mechanisms. The view that mutated oncogenes such as KRAS or PIK3CA are only hyperactive proteins must be modified, as several studies have described additional activities suggestive of neomorphic (GOF) activity, but as these observations were based on cell transfection with protein overexpression more studies are needed to assess the exact behavior of all of these genes.^97, 98^ The boundaries between hypermorph and neomorph mutations are not clear cut and alterations in a single gene can lead to variants that can have either one or both characteristics.

Although chromosomal translocations leading to fusion genes and the synthesis of chimeric proteins with novel functional specificity are the most obvious and easy-to-demonstrate neomorph mutations, it is more difficult to demonstrate missense mutations acting as neomorph mutations. Nevertheless, neomorphic mutations have recently been demonstrated in isocitrate dehydrogenases 1 and 2 (IDH1 and IDH2), which are mutated in several cancer types such as glioma and AML (Table 1). In both genes, a single missense mutation affecting an amino acid residue localized in the catalytic region of the protein accounted for >90% of reported events and were shown to change the substrate specificity of these enzymes.^99^ As discussed in the previous section, several hotspot p53 mutants are obviously neomorphic with a marked diversity in the activity gained by each variant.

## Conclusion

*In vitro*, mouse *in vivo* and clinical studies all point toward the importance of selection for oncogenic p53 mutants in human tumors. Although classification of cancer genes into oncogenes or tumor suppressor genes can be very helpful, it is becoming increasingly obvious that the frontier between the two classes is not as clear as previously thought, and that some genes may straddle these two categories. Furthermore, tissue type, genetic background and many other factors also influence the phenotype induced by a specific mutation. Nonetheless, we can predict that, except for rare cases, most cancer gene alterations will give rise to products with pleiotropic activities. Using the classification based on Muller's proposition more than 80 years ago, it is possible to establish a comprehensive and meaningful categorization of the various cancer genes that illustrates how TP53 does not fit the classical definition of a tumor suppressor gene. Beyond the rhetorical aspect of this statement, there is a genuine need to avoid an overly simplistic, binary, ‘wild-type *versus* inactivated' classification; defining p53 mutants as oncogenes with heterogeneous GOFs that affect multiple pathways must be considered. Designing drugs to target specific p53 mutants seems an attractive strategy, as several of these mutants are among the most frequent protein variants found in several cancer types and associated with the death of >120,000 patients worldwide. Indeed, small molecules capable of targeting hotspot mutants p.R175H or p.Y220C have been identified. Further drug discovery efforts should focus on other frequent p53 mutants, such as p.R273H, p.R248W and p.R249S. With drugs targeting a wide range of common p53 mutants in our future arsenal, our chances of efficiently fighting cancer may be greatly improved.^100^

## Figures and Tables

**Table 1:** Classification of cancer mutations according to their consequences on protein activity

	Amorph	Hypomorph	Antimorph	Hypermorph	Neomorph	Mutation type
**TP53**	Yes	Yes	Yes	Yes	Yes	Mis, N, F
**Rb1**	Yes	Yes	No	No	No	D, Mis, N, F, S
**CDKN2A**	Yes	No	No	No	No	D, Mis, N, F, S, Met
**PTEN**	Yes	No	Yes	No	No	D, Mis, N, F, S
**MLH1**	Yes	No	No	No	No	D, Mis, N, F, S, Met
**FBXW7**	Yes	No	No	No	No	Mis, N, D, F
**GATA3**	Yes	No	No	No	No	F, N, S
**KDM6A**	Yes	No	No	No	No	D, N, F, S
**STAG2**	Yes	No	No	No	No	Mis, N, F, S
**STK11**	Yes	No	No	No	No	D, Mis, N, F, S
**RPL22**	Yes	No	No	No	No	F
**RUNX1**	Yes	No	No	No	No	T, Mis
**APC**	Yes	Yes	No	No	No	D, Mis, N, F, S, Met
**NF1**	Yes	No	No	No	No	D, Mis, N, F, S
**ARID1A**	Yes	No	No	No	No	N, Mis, F, S, D
**BAP1**	Yes	No	No	No	No	N, Mis, F, S, O
**CDH1**	Yes	No	No	No	No	Mis, N, F, S
**MAP2K4**	Yes	No	No	No	No	D, Mis, N
**MLL3**	Yes	No	No	No	No	Mis, S, F
**PBRM1**	Yes	No	No	No	No	Mis, N, F, S, D, O
**BRCA1**	Yes	Yes	No	No	No	D, Mis, N, F, S, Met
**VHL**	Yes	Yes	No	No	No	D, Mis, N, F, S
**APC**	Yes	Yes	No	No	No	D, Mis, N, F, S, Met
**RET**	Yes	Yes	No	Yes	No	T, Mis, N, F
**DNMT3A**	Yes	No	Yes	No	No	Mis, F, N, S
**NOTCH1**	Yes	No	No	Yes	No	T, Mis, O
**BRAF**	No	No	No	Yes	No	Mis, T, O
**KRAS**	No	No	No	Yes	No	Mis
**MYC**	No	No	No	Yes	No	A, T
**MDM2**	No	No	No	Yes	No	A
**AKT1**	No	No	No	Yes	No	Mis
**MYCN**	No	No	No	Yes	No	A
**CTNNB1**	No	No	No	Yes	No	Mis, T
**EGFR**	No	No	No	Yes	No	A, O, Mis
**MALAT1**	No	No	No	Yes	No	T Amp
**NFE2L2**	No	No	No	Yes	No	Mis
**NPM1**	No	No	No	Yes	No	T, F
**NRAS**	No	No	No	Yes	No	Mis
**PIK3CA**	No	No	No	Yes	No	Mis
**PIK3R1**	No	No	No	Yes	Yes	Mis, F, O
**SETBP1**	No	No	No	Yes	No	Mis
**IDH1**	No	No	No	No	Yes	Mis
**IDH2**	No	No	No	No	Yes	Mis
**FLT3**	No	No	No	No	Yes	Mis, O

Abbreviations: D, gene deletion; F, frameshift mutation (small insertion and deletion); Met, loss of expression via promoter hypermethylation; Mis, missense mutation; N, nonsense mutation; O, other (splice mutations, intronic mutation); S, synonymous mutation; T, gene translocation

Mutation type for each cancer gene was extracted from the cancer gene census at the COSMIC database except for the methylation status (http://cancer.sanger.ac.uk/cancergenome/projects/cell_lines/). Fusion proteins generated via chromosomal translocation are not included in this list

## Abbreviations

LOH: loss of heterozygosity
LOF: loss-of-function
GOF: gain-of-function
AML: acute myeloid leukemia
CHIP: C-terminus of Hsc70 Interacting Protein
MQ: methylene quinuclidinone
RETRA: reactivation of transcriptional reporter activity
KRAS: v-Ki-ras2 Kirsten rat sarcoma 2 viral oncogene homolog
IDH1: isocitrate dehydrogenase 1
PIK3CA: phosphoinositide-3-kinase, catalytic, alpha polypeptide
TP53: tumor protein p53
PTEN: phosphatase and tensin homolog gene
APC: adenomatous polyposis coli gene

## Notes

KGW is a co-founder, shareholder and board member of the company Aprea AB that develops p53-based cancer therapy, including APR-246. TS declares no conflict of interest.

## References

[bib1] Garraway LA Lander ES Lessons from the cancer genome Cell 2013 153 17 37 23540688 10.1016/j.cell.2013.03.002

[bib2] Koboldt DC Steinberg KM Larson DE Wilson RK Mardis ER The next-generation sequencing revolution and its impact on genomics Cell 2013 155 27 38 24074859 10.1016/j.cell.2013.09.006 PMC3969849

[bib3] Kandoth C McLellan MD Vandin F Ye K Niu B Lu C Mutational landscape and significance across 12 major cancer types Nature 2013 502 333 339 24132290 10.1038/nature12634 PMC3927368

[bib4] Vogelstein B Papadopoulos N Velculescu VE Zhou S Diaz LAJ Kinzler KW Cancer genome landscapes Science 2013 339 1546 1558 23539594 10.1126/science.1235122 PMC3749880

[bib5] Lawrence MS Stojanov P Polak P Kryukov GV Cibulskis K Sivachenko A Mutational heterogeneity in cancer and the search for new cancer-associated genes Nature 2013 499 214 218 23770567 10.1038/nature12213 PMC3919509

[bib6] Stratton MR Exploring the genomes of cancer cells: progress and promise Science 2011 331 1553 1558 21436442 10.1126/science.1204040

[bib7] Workman P Al-Lazikani B Drugging cancer genomes Nat Rev Drug Discov 2013 12 889 890 24287764 10.1038/nrd4184

[bib8] Harris H Miller OJ Klein G Worst P Tachibana T Suppression of malignancy by cell fusion Nature 1969 223 363 368 5387828 10.1038/223363a0

[bib9] Knudson A Mutation and cancer: statistical study of retinoblastoma Proc Natl Acad Sci USA 1971 68 820 823 5279523 10.1073/pnas.68.4.820 PMC389051

[bib10] Cavenee WK Dryja TP Phillips RA Benedict WF Godbout R Gallie BL Expression of recessive alleles by chromosomal mechanisms in retinoblastoma Nature 1983 305 779 784 6633649 10.1038/305779a0

[bib11] Haber D Harlow E Tumour-suppressor genes: evolving definitions in the genomic age Nat Genet 1997 16 320 322 9241260 10.1038/ng0897-320

[bib12] Kinzler KW Vogelstein B Cancer-susceptibility genes. Gatekeepers and caretakers Nature 1997 386 763 10.1038/386761a0 9126728

[bib13] Weng AP Ferrando AA Lee W Morris JP Silverman LB Sanchez-Irizarry C Activating mutations of NOTCH1 in human T cell acute lymphoblastic leukemia Science 2004 306 269 271 15472075 10.1126/science.1102160

[bib14] Nicolas M Wolfer A Raj K Kummer JA Mill P van Noort Notch1 functions as a tumor suppressor in mouse skin Nat Genet 2003 33 416 421 12590261 10.1038/ng1099

[bib15] Baker SJ Fearon ER Nigro JM Hamilton SR Preisinger AC Jessup JM Chromosome 17 deletions and p53 gene mutations in colorectal carcinomas Science 1989 244 217 221 2649981 10.1126/science.2649981

[bib16] Takahashi T Nau MM Chiba I Birrer MJ Rosenberg RK Vinocour p53: a frequent target for genetic abnormalities in lung cancer Science 1989 246 491 494 2554494 10.1126/science.2554494

[bib17] Soussi T Wiman KG Shaping genetic alterations in human cancer: the p53 mutation paradigm Cancer Cell 2007 12 303 312 17936556 10.1016/j.ccr.2007.10.001

[bib18] Freed-Pastor WA Prives C Mutant p53: one name, many proteins Genes Dev 2012 26 1268 1286 22713868 10.1101/gad.190678.112 PMC3387655

[bib19] Leroy B Anderson M Soussi T TP53 mutations in human cancer: database reassessment and prospects for the next decade Hum Mutat 2014 35 672 688 24665023 10.1002/humu.22552

[bib20] Soussi T Advances in carcinogenesis: a historical perspective from observational studies to tumor genome sequencing and TP53 mutation spectrum analysis Biochim Biophys Acta 2011 1816 199 208 21791238 10.1016/j.bbcan.2011.07.003

[bib21] Joerger AC Fersht AR The tumor suppressor p53: from structures to drug discovery Cold Spring Harb Perspect Biol 2010 2 a000919 20516128 10.1101/cshperspect.a000919 PMC2869527

[bib22] Weinstein JN Collisson EA Mills GB Shaw KR Ozenberger BA Ellrott K The Cancer Genome Atlas Pan-Cancer analysis project Nat Genet 2013 45 1113 1120 24071849 10.1038/ng.2764 PMC3919969

[bib23] Bartholomeusz C Gonzalez-Angulo AM Targeting the PI3K signaling pathway in cancer therapy Expert Opin Ther Targets 2012 16 121 130 22239433 10.1517/14728222.2011.644788

[bib24] Aas T Borresen AL Geisler S Smith-Sorensen B Johnsen H Varhaug JE Specific P53 mutations are associated with de novo resistance to doxorubicin in breast cancer patients Nat Med 1996 2 811 814 8673929 10.1038/nm0796-811

[bib25] Zenz T Vollmer D Trbusek M Smardova J Benner A Soussi T TP53 mutation profile in chronic lymphocytic leukemia: evidence for a disease specific profile from a comprehensive analysis of 268 mutations Leukemia 2010 24 2072 2079 20861914 10.1038/leu.2010.208

[bib26] Bartek J Bartkova J Vojtesek B Staskova Z Lukas J Rejthar A Aberrant expression of the p53 oncoprotein is a common feature of a wide spectrum of human malignancies Oncogene 1991 6 1699 1703 1923535

[bib27] Benchimol S Pim D Crawford L Radioimmunoassay of the cellular protein p53 in mouse and human cell lines EMBO J 1982 1 1055 1062 6765238 10.1002/j.1460-2075.1982.tb01296.x PMC553162

[bib28] Terzian T Suh YA Iwakuma T Post SM Neumann M Lang GA The inherent instability of mutant p53 is alleviated by Mdm2 or p16INK4a loss Genes Dev 2008 22 1337 1344 18483220 10.1101/gad.1662908 PMC2377188

[bib29] Hock AK Vousden KH The role of ubiquitin modification in the regulation of p53 Biochim Biophys Acta 2014 1843 137 149 23742843 10.1016/j.bbamcr.2013.05.022

[bib30] Pant V Lozano G Limiting the power of p53 through the ubiquitin proteasome pathway Genes Dev 2014 28 1739 1751 25128494 10.1101/gad.247452.114 PMC4197966

[bib31] Li D Marchenko ND Schulz R Fischer V Velasco-Hernandez T Talos F Functional inactivation of endogenous MDM2 and CHIP by HSP90 causes aberrant stabilization of mutant p53 in human cancer cells Mol Cancer Res 2011 9 577 588 21478269 10.1158/1541-7786.MCR-10-0534 PMC3097033

[bib32] Gannon JV Greaves R Iggo R Lane DP Activating mutations in p53 produce a common conformational effect. A monoclonal antibody specific for the mutant form EMBO J 1990 9 1595 1602 1691710 10.1002/j.1460-2075.1990.tb08279.x PMC551855

[bib33] Hinds PW Finlay CA Quartin RS Baker SJ Fearon ER Vogelstein B Mutant p53 DNA clones from human colon carcinomas cooperate with ras in transforming primary rat cells: a comparison of the ‘hot spot' mutant phenotypes Cell Growth Differ 1990 1 571 580 2288874

[bib34] Ory K Legros Y Auguin C Soussi T Analysis of the most representative tumour-derived p53 mutants reveals that changes in protein conformation are not correlated with loss of transactivation or inhibition of cell proliferation EMBO J 1994 13 3496 3504 8062826 10.1002/j.1460-2075.1994.tb06656.x PMC395253

[bib35] Milner J A conformation hypothesis for the suppressor and promoter functions of p53 in cell growth control and in cancer Proc Biol Sci 1991 245 139 145 1682937 10.1098/rspb.1991.0100

[bib36] Wong KB DeDecker BS Freund SM Proctor MR Bycroft M Fersht AR Hot-spot mutants of p53 core domain evince characteristic local structural changes Proc Natl Acad Sci USA 1999 96 8438 8442 10411893 10.1073/pnas.96.15.8438 PMC17534

[bib37] Bullock AN Henckel J DeDecker BS Johnson CM Nikolova PV Proctor MR Thermodynamic stability of wild-type and mutant p53 core domain Proc Natl Acad Sci USA 1997 94 14338 14342 9405613 10.1073/pnas.94.26.14338 PMC24967

[bib38] Cho Y Gorina S Jeffrey PD Pavletich NP Crystal structure of a p53 tumor suppressor-DNA complex: understanding tumorigenic mutations Science 1994 265 346 355 8023157 10.1126/science.8023157

[bib39] Halevy O Michalovitz D Oren M Different tumor-derived p53 mutants exhibit distinct biological activities Science 1990 250 113 116 2218501 10.1126/science.2218501

[bib40] Kawaguchi T Kato S Otsuka K Watanabe G Kumabe T Tominaga T The relationship among p53 oligomer formation, structure and transcriptional activity using a comprehensive missense mutation library Oncogene 2005 24 6976 6981 16007150 10.1038/sj.onc.1208839

[bib41] Kakudo Y Shibata H Otsuka K Kato S Ishioka C Lack of correlation between p53-dependent transcriptional activity and the ability to induce apoptosis among 179 mutant p53s Cancer Res 2005 65 2108 2114 15781620 10.1158/0008-5472.CAN-04-2935

[bib42] Shiraishi K Kato S Han SY Liu W Otsuka K Sakayori Isolation of temperature-sensitive p53 mutations from a comprehensive missense mutation library J Biol Chem 2004 279 348 355 14559903 10.1074/jbc.M310815200

[bib43] Kato S Han SY Liu W Otsuka K Shibata H Kanamaru R Understanding the function-structure and function-mutation relationships of p53 tumor suppressor protein by high-resolution missense mutation analysis Proc Natl Acad Sci USA 2003 100 8424 8429 12826609 10.1073/pnas.1431692100 PMC166245

[bib44] Oren M Rotter V Mutant p53 gain-of-function in cancer Cold Spring Harb Perspect Biol 2010 2 a001107 20182618 10.1101/cshperspect.a001107 PMC2828285

[bib45] Muller PA Vousden KH p53 mutations in cancer Nat Cell Biol 2013 15 2 8 23263379 10.1038/ncb2641

[bib46] Donzelli S Biagioni F Fausti F Strano S Fontemaggi G Blandino G Oncogenomic approaches in exploring gain of function of mutant p53 Curr Genomics 2008 9 200 207 19440517 10.2174/138920208784340713 PMC2679646

[bib47] Bieging KT Mello SS Attardi LD Unravelling mechanisms of p53-mediated tumour suppression Nat Rev Cancer 2014 14 359 370 24739573 10.1038/nrc3711 PMC4049238

[bib48] Dittmer D Pati S Zambetti G Chu S Teresky AK Moore Gain of function mutations in p53 Nat Genet 1993 4 42 46 8099841 10.1038/ng0593-42

[bib49] Ciriello G Miller ML Aksoy BA Senbabaoglu Y Schultz N Sander C Emerging landscape of oncogenic signatures across human cancers Nat Genet 2013 45 1127 1133 24071851 10.1038/ng.2762 PMC4320046

[bib50] Stambolsky P Tabach Y Fontemaggi G Weisz L Maor-Aloni R Siegfried Z Modulation of the vitamin D3 response by cancer-associated mutant p53 Cancer Cell 2010 17 273 285 20227041 10.1016/j.ccr.2009.11.025 PMC2882298

[bib51] Dell'orso S Fontemaggi G Stambolsky P Goeman F Voellenkle C Levrero ChIP-on-Chip analysis of *in vivo* mutant p53 binding to selected gene promoters OMICS 2011 15 305 312 21332394 10.1089/omi.2010.0084

[bib52] Dong P Karaayvaz M Jia N Kaneuchi M Hamada J Watari H Mutant p53 gain-of-function induces epithelial-mesenchymal transition through modulation of the miR-130b-ZEB1 axis Oncogene 2013 32 3286 3295 22847613 10.1038/onc.2012.334 PMC3705163

[bib53] Santoro R Strano S Blandino G Transcriptional regulation by mutant p53 and oncogenesis Subcell Biochem 2014 85 91 103 25201190 10.1007/978-94-017-9211-0_5

[bib54] Zhou G Wang J Zhao M Xie TX Tanaka N Sano D Gain-of-function mutant p53 promotes cell growth and cancer cell metabolism via inhibition of AMPK activation Mol Cell 2014 54 960 974 24857548 10.1016/j.molcel.2014.04.024 PMC4067806

[bib55] Zhang C Liu J Liang Y Wu R Zhao Y Hong X Tumour-associated mutant p53 drives the Warburg effect Nat Commun 2013 4 2935 24343302 10.1038/ncomms3935 PMC3969270

[bib56] Yang A Kaghad M Wang Y Gillett E Fleming MD Dotsch V p63, a p53 homolog at 3q27-29, encodes multiple products with transactivating, death-inducing, and dominant-negative activities Mol Cell 1998 2 305 316 9774969 10.1016/s1097-2765(00)80275-0

[bib57] Kaghad M Bonnet H Yang A Creancier L Biscan JC Valent A Monoallelically expressed gene related to p53 at 1p36, a region frequently deleted in neuroblastoma and other human cancers Cell 1997 90 809 819 9288759 10.1016/s0092-8674(00)80540-1

[bib58] Di Como CJ Gaiddon C Prives C p73 function is inhibited by tumor-derived p53 mutants in mammalian cells Mol Cell Biol 1999 19 1438 1449 9891077 10.1128/mcb.19.2.1438 PMC116072

[bib59] Bergamaschi D Gasco M Hiller L Sullivan A Syed N Trigiante G p53 polymorphism influences response in cancer chemotherapy via modulation of p73-dependent apoptosis Cancer Cell 2003 3 387 402 12726864 10.1016/s1535-6108(03)00079-5

[bib60] Gaiddon C Lokshin M Ahn J Zhang T Prives C A subset of tumor-derived mutant forms of p53 down-regulate p63 and p73 through a direct interaction with the p53 core domain Mol Cell Biol 2001 21 1874 1887 11238924 10.1128/MCB.21.5.1874-1887.2001 PMC86759

[bib61] Li Y Prives C Are interactions with p63 and p73 involved in mutant p53 gain of oncogenic function Oncogene 2007 26 2220 2225 17401431 10.1038/sj.onc.1210311

[bib62] Li T Kon N Jiang L Tan M Ludwig T Zhao Y Tumor suppression in the absence of p53-mediated cell-cycle arrest, apoptosis, and senescence Cell 2012 149 1269 1283 22682249 10.1016/j.cell.2012.04.026 PMC3688046

[bib63] Brady CA Jiang D Mello SS Johnson TM Jarvis LA Kozak MM Distinct p53 transcriptional programs dictate acute DNA-damage responses and tumor suppression Cell 2011 145 571 583 21565614 10.1016/j.cell.2011.03.035 PMC3259909

[bib64] Valente LJ Gray DH Michalak EM Pinon-Hofbauer J Egle A Scott CL p53 efficiently suppresses tumor development in the complete absence of its cell-cycle inhibitory and proapoptotic effectors p21, Puma, and Noxa Cell Rep 2013 3 1339 1345 23665218 10.1016/j.celrep.2013.04.012

[bib65] Hock AK Vousden KH Tumor suppression by p53: fall of the triumvirate Cell 2012 149 1183 1185 22682240 10.1016/j.cell.2012.05.024

[bib66] Donehower LA Harvey M Slagle BL McArthur MJ Montgomery CAJ Butel JS Mice deficient for p53 are developmentally normal but susceptible to spontaneous tumours Nature 1992 356 215 221 1552940 10.1038/356215a0

[bib67] Donehower LA Lozano G 20 years studying p53 functions in genetically engineered mice Nat Rev Cancer 2009 9 831 841 19776746 10.1038/nrc2731

[bib68] Kenzelmann Broz D Attardi LD *In vivo* analysis of p53 tumor suppressor function using genetically engineered mouse models Carcinogenesis 2010 31 1311 1318 20097732 10.1093/carcin/bgp331 PMC2915627

[bib69] Lang GA Iwakuma T Suh YA Liu G Rao VA Parant JM Gain of function of a p53 hot spot mutation in a mouse model of Li-Fraumeni syndrome Cell 2004 119 861 872 15607981 10.1016/j.cell.2004.11.006

[bib70] Olive KP Tuveson DA Ruhe ZC Yin B Willis NA Bronson RT Mutant p53 gain of function in two mouse models of Li-Fraumeni syndrome Cell 2004 119 847 860 15607980 10.1016/j.cell.2004.11.004

[bib71] Ludwig RL Bates S Vousden KH Differential activation of target cellular promoters by p53 mutants with impaired apoptotic function Mol Cell Biol 1996 16 4952 4960 8756654 10.1128/mcb.16.9.4952 PMC231497

[bib72] Liu G Parant JM Lang G Chau P Chavez-Reyes A El-Naggar AK Chromosome stability, in the absence of apoptosis, is critical for suppression of tumorigenesis in Trp53 mutant mice Nat Genet 2004 36 63 68 14702042 10.1038/ng1282

[bib73] Khoo KH Verma CS Lane DP Drugging the p53 pathway: understanding the route to clinical efficacy Nat Rev Drug Discov 2014 13 217 236 24577402 10.1038/nrd4236

[bib74] Bykov VJ Wiman KG Mutant p53 reactivation by small molecules makes its way to the clinic FEBS Lett 2014 588 2622 2627 24768524 10.1016/j.febslet.2014.04.017

[bib75] Wilcken R Wang G Boeckler FM Fersht AR Kinetic mechanism of p53 oncogenic mutant aggregation and its inhibition Proc Natl Acad Sci USA 2012 109 13584 13589 22869713 10.1073/pnas.1211550109 PMC3427094

[bib76] Boeckler FM Joerger AC Jaggi G Rutherford TJ Veprintsev DB Fersht AR Targeted rescue of a destabilized mutant of p53 by an in silico screened drug Proc Natl Acad Sci USA 2008 105 10360 10365 18650397 10.1073/pnas.0805326105 PMC2492497

[bib77] Liu X Wilcken R Joerger AC Chuckowree IS Amin J Spencer J Small molecule induced reactivation of mutant p53 in cancer cells Nucleic Acids Res 2013 41 6034 6044 23630318 10.1093/nar/gkt305 PMC3695503

[bib78] Yu X Vazquez A Levine AJ Carpizo DR Allele-specific p53 mutant reactivation Cancer Cell 2012 21 614 625 22624712 10.1016/j.ccr.2012.03.042 PMC3366694

[bib79] Yu X Blanden AR Narayanan S Jayakumar L Lubin D Augeri D Small molecule restoration of wildtype structure and function of mutant p53 using a novel zinc-metallochaperone based mechanism Oncotarget 2014 5 8879 8892 25294809 10.18632/oncotarget.2432 PMC4253404

[bib80] Bykov VJ Issaeva N Shilov A Hultcrantz M Pugacheva E Chumakov P Restoration of the tumor suppressor function to mutant p53 by a low-molecular-weight compound Nat Med 2002 8 282 288 11875500 10.1038/nm0302-282

[bib81] Bykov VJ Issaeva N Zache N Shilov A Hultcrantz M Bergman J Reactivation of mutant p53 and induction of apoptosis in human tumor cells by maleimide analogs J Biol Chem 2005 280 30384 30391 15998635 10.1074/jbc.M501664200

[bib82] Bykov VJ Zache N Stridh H Westman J Bergman J Selivanova G PRIMA-1(MET) synergizes with cisplatin to induce tumor cell apoptosis Oncogene 2005 24 3484 3491 15735745 10.1038/sj.onc.1208419

[bib83] Lambert JM Moshfegh A Hainaut P Wiman KG Bykov VJ Mutant p53 reactivation by PRIMA-1(MET) induces multiple signaling pathways converging on apoptosis Oncogene 2009 29 1329 1338 19946333 10.1038/onc.2009.425

[bib84] Wassman CD Baronio R Demir O Wallentine BD Chen CK Hall LV Computational identification of a transiently open L1/S3 pocket for reactivation of mutant p53 Nat Commun 2013 4 1407 23360998 10.1038/ncomms2361 PMC3562459

[bib85] Lehmann S Bykov VJ Ali D Andren O Cherif H Tidefelt U Targeting p53 *in vivo*: a first-in-human study with p53-targeting compound APR-246 in refractory hematologic malignancies and prostate cancer J Clin Oncol 2012 30 3633 3639 22965953 10.1200/JCO.2011.40.7783

[bib86] Peng X Zhang MQ Conserva F Hosny G Selivanova G Bykov VJ APR-246/PRIMA-1MET inhibits thioredoxin reductase 1 and converts the enzyme to a dedicated NADPH oxidase Cell Death Dis 2013 4 e881 24157875 10.1038/cddis.2013.417 PMC3920950

[bib87] Tessoulin B Descamps G Moreau P Maiga S Lode L Godon C PRIMA-1Met induces myeloma cell death independent of p53 by impairing the GSH/ROS balance Blood 2014 124 1626 1636 25006124 10.1182/blood-2014-01-548800

[bib88] Kravchenko JE Ilyinskaya GV Komarov PG Agapova LS Kochetkov DV Strom E Small-molecule RETRA suppresses mutant p53-bearing cancer cells through a p73-dependent salvage pathway Proc Natl Acad Sci USA 2008 105 6302 6307 18424558 10.1073/pnas.0802091105 PMC2327210

[bib89] Floquet C Deforges J Rousset JP Bidou L Rescue of non-sense mutated p53 tumor suppressor gene by aminoglycosides Nucleic Acids Res 2011 39 3350 3362 21149266 10.1093/nar/gkq1277 PMC3082906

[bib90] Muller HJ Further studies on the nature and causes of gene mutations Int Congr Genet 1932 6 231 255

[bib91] Cox C Bignell G Greenman C Stabenau A Warren W Stephens P A survey of homozygous deletions in human cancer genomes Proc Natl Acad Sci USA 2005 102 4542 4547 15761058 10.1073/pnas.0408593102 PMC555487

[bib92] Rowan AJ Lamlum H Ilyas M Wheeler J Straub J Papadopoulou A APC mutations in sporadic colorectal tumors: A mutational ‘hotspot' and interdependence of the ‘two hits' Proc Natl Acad Sci USA 2000 97 3352 3357 10737795 10.1073/pnas.97.7.3352 PMC16243

[bib93] Herskowitz I Functional inactivation of genes by dominant negative mutations Nature 1987 329 219 222 2442619 10.1038/329219a0

[bib94] Natan E Hirschberg D Morgner N Robinson CV Fersht AR Ultraslow oligomerization equilibria of p53 and its implications Proc Natl Acad Sci USA 2009 106 14327 14332 19667193 10.1073/pnas.0907840106 PMC2731847

[bib95] Wang Y Suh YA Fuller MY Jackson JG Xiong S Terzian T Restoring expression of wild-type p53 suppresses tumor growth but does not cause tumor regression in mice with a p53 missense mutation J Clin Invest 2011 121 893 904 21285512 10.1172/JCI44504 PMC3049366

[bib96] Scuoppo C Miething C Lindqvist L Reyes J Ruse C Appelmann I A tumour suppressor network relying on the polyamine-hypusine axis Nature 2012 487 244 248 22722845 10.1038/nature11126 PMC3530829

[bib97] Gymnopoulos M Elsliger MA Vogt PK Rare cancer-specific mutations in PIK3CA show gain of function Proc Natl Acad Sci USA 2007 104 5569 5574 17376864 10.1073/pnas.0701005104 PMC1838453

[bib98] Cirstea IC Gremer L Dvorsky R Zhang SC Piekorz RP Zenker Diverging gain-of-function mechanisms of two novel KRAS mutations associated with Noonan and cardio-facio-cutaneous syndromes Hum Mol Genet 2013 22 262 270 23059812 10.1093/hmg/dds426

[bib99] Dang L White DW Gross S Bennett BD Bittinger MA Driggers EM Cancer-associated IDH1 mutations produce 2-hydroxyglutarate Nature 2010 465 966 20559394 10.1038/nature09132 PMC3766976

[bib100] Muller PA Vousden KH Mutant p53 in cancer: new functions and therapeutic opportunities Cancer Cell 2014 25 304 317 24651012 10.1016/j.ccr.2014.01.021 PMC3970583

