# Supplementary material for: TP53: an oncogene in disguise
Source: Cell Death Differ. 2015 May 29;22(8):1239–49. doi: 10.1038/cdd.2015.53 (PMC4495363; doi:10.1038/cdd.2015.53)
Supplement: Supplementary Figure 1 [file cdd201553x1.pdf]

TP53: an oncogene in disguise

Thierry Soussi and Klas G. Wiman

Supplementary Figure 1:
Left panel: Genes most frequently mutated in all types of cancer in the Pan-Cancer study. Only the top six genes are shown in the graph. Right panel: most frequent protein variants in all types of cancer in the Pan-Cancer study. Left Y axis: mutant variant frequency; Right Y axis: number of worldwide cancer cases associated with the different variants.
A: analysis was performed with the twelve types of cancer included in the PAN-CANCER study.
B to G: same analysis for each type of cancer of the PAN-CANCER study.

Data were generated by analysis of the mutations released by the Cancer Genome Atlas (TCGA) (http://www.cbioportal.org. and http://cbio.mskcc.org/index.html). Cancer cases were estimated using year 2008 data from Globocan (http://globocan.iarc.fr/).

A **Pan-Cancer**

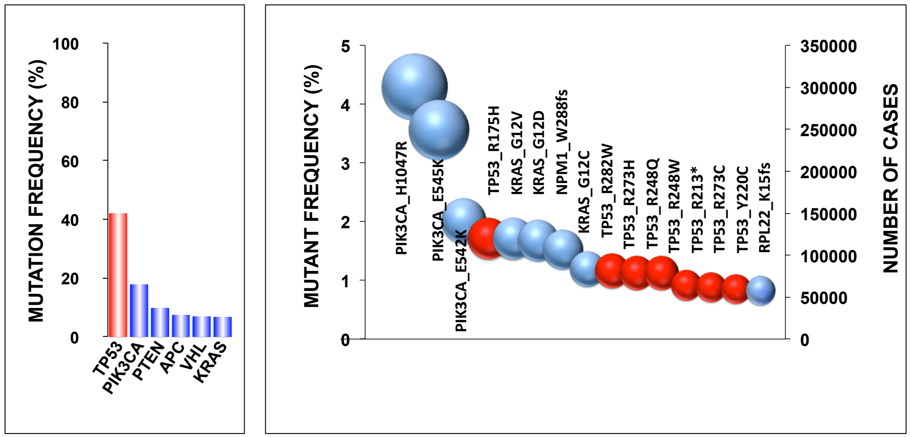

B **OV**

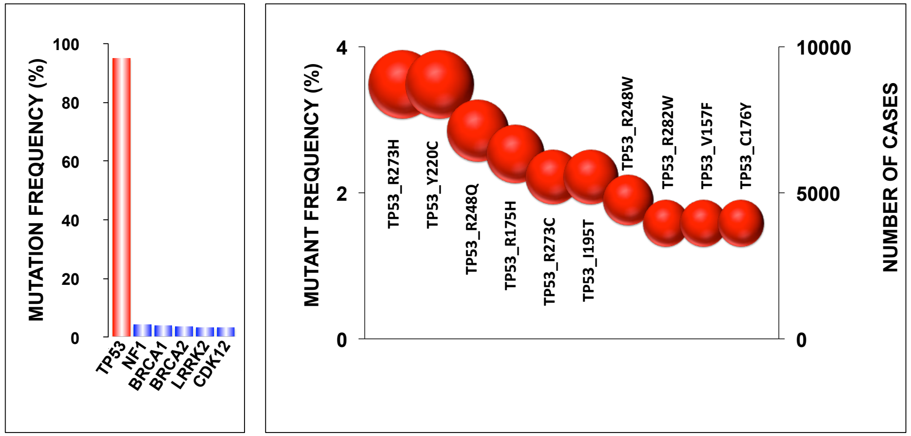

C **LUSC**

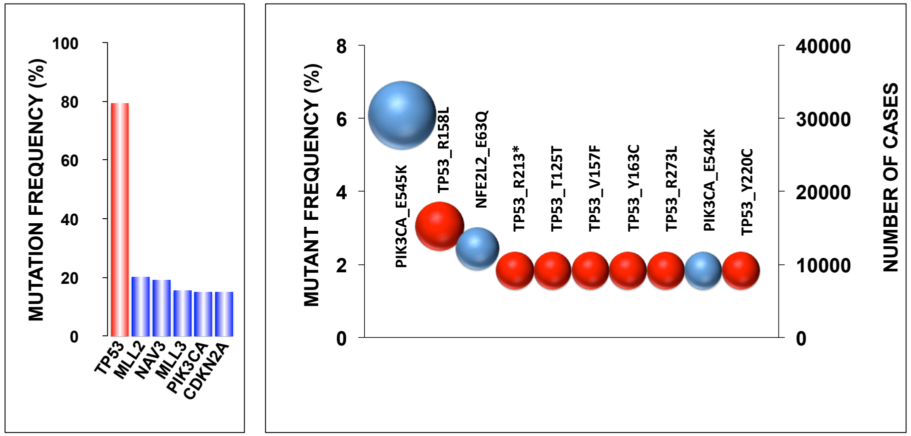

D

HNSC

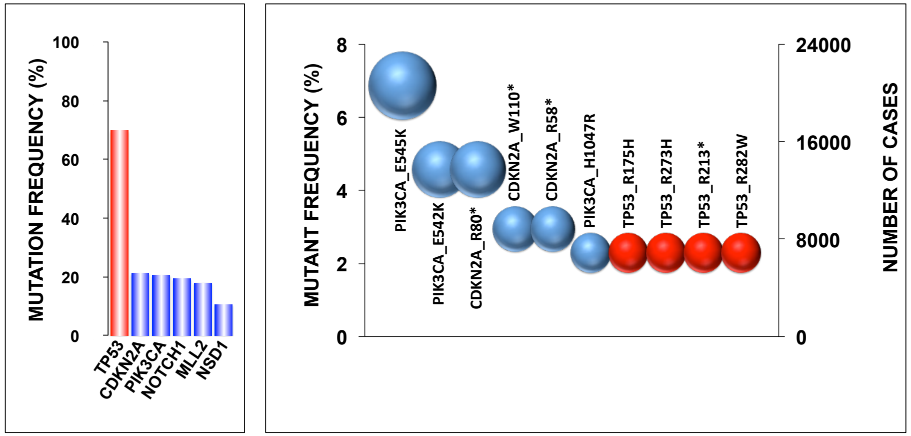

E

COAD/READ

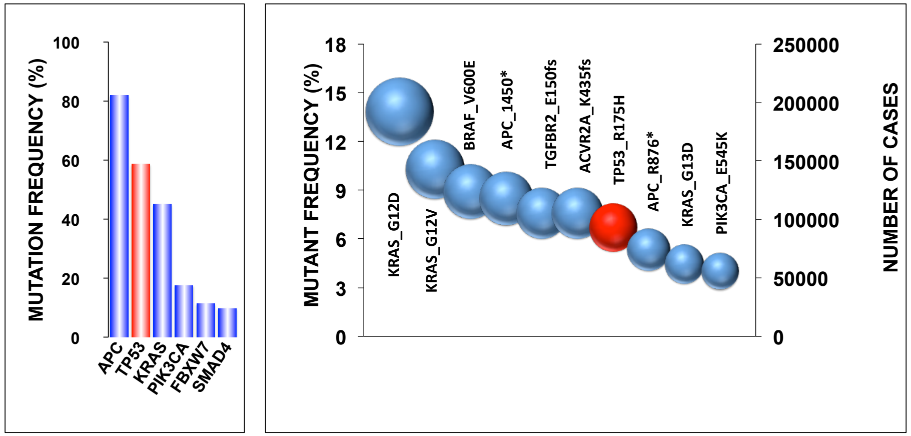

F

LUAD

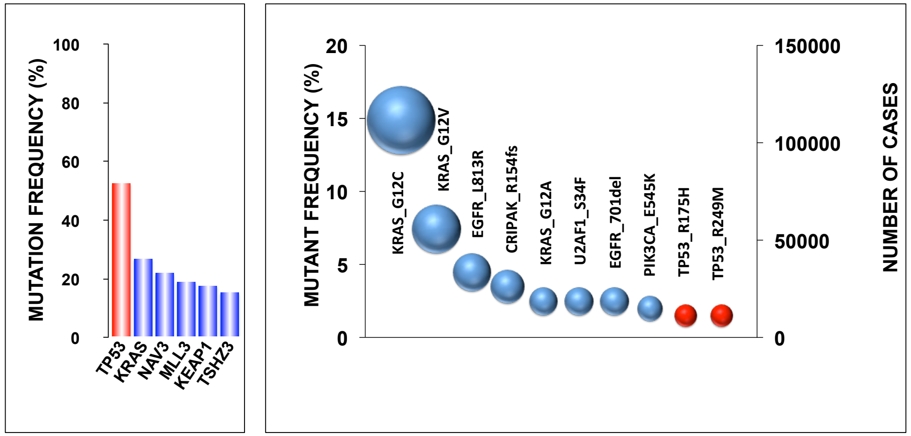

G

LUCA

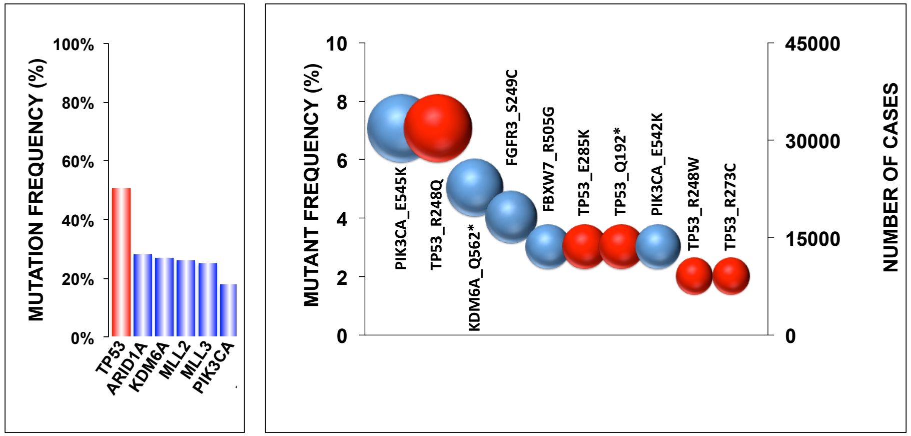

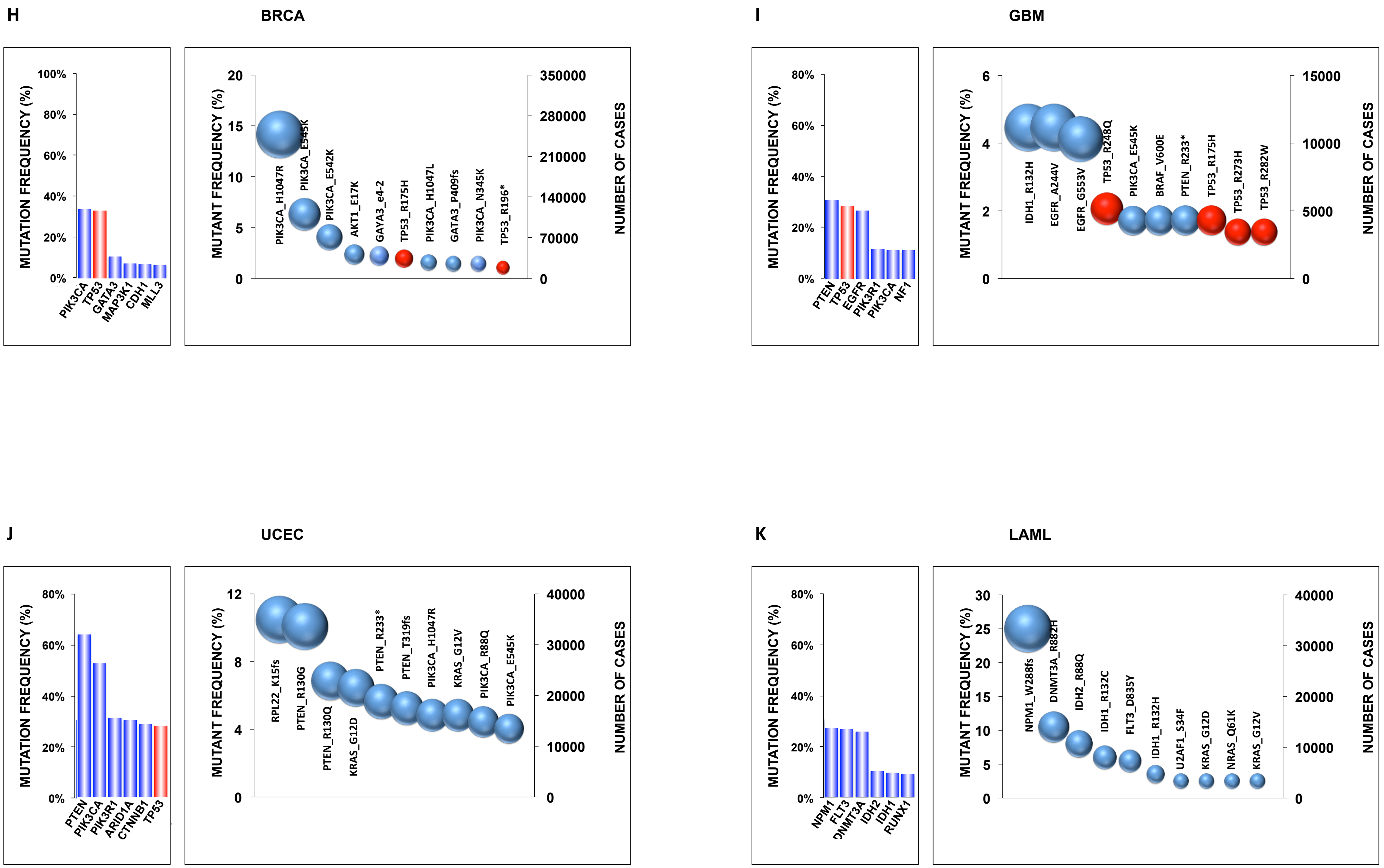
H
BRCA
MUTATION FREQUENCY (%)
100%
80%
60%
40%
20%
0%
PIK3CA
TP53
GATA3
MAP3K1
CDH1
MLL3
MUTANT FREQUENCY (%)
20
15
10
5
0
NUMBER OF CASES
350000
280000
210000
140000
70000
0
PIK3CA_H1047R
PIK3CA_E545K
PIK3CA_E542K
AKT1_E17K
GAYA3_e4-2
TP53_R175H
PIK3CA_H1047L
GATA3_P409fs
PIK3CA_N345K
TP53_R196*
I
GBM
MUTATION FREQUENCY (%)
80%
60%
40%
20%
0%
PTEN
TP53
EGFR
PIK3R1
PIK3CA
NF1
MUTANT FREQUENCY (%)
6
4
2
0
NUMBER OF CASES
15000
10000
5000
0
IDH1_R132H
EGFR_A244V
EGFR_G553V
TP53_R248Q
PIK3CA_E545K
BRAF_V600E
PTEN_R233*
TP53_R175H
TP53_R273H
TP53_R282W
J
UCEC
MUTATION FREQUENCY (%)
80%
60%
40%
20%
0%
PTEN
PIK3CA
PIK3R1
ARID1A
CTNNB1
TP53
MUTANT FREQUENCY (%)
12
8
4
0
NUMBER OF CASES
40000
30000
20000
10000
0
RPL22_K15fs
PTEN_R130G
PTEN_R130Q
KRAS_G12D
PTEN_R233*
PTEN_T319fs
PIK3CA_H1047R
KRAS_G12V
PIK3CA_R88Q
PIK3CA_E545K
K
LAML
MUTATION FREQUENCY (%)
80%
60%
40%
20%
0%
NPM1
FLT3
DNMT3A
IDH2
IDH1
RUNX1
MUTANT FREQUENCY (%)
30
25
20
15
10
5
0
NUMBER OF CASES
40000
30000
20000
10000
0
NPM1_W288fs
DNMT3A_R882H
IDH2_R88Q
IDH1_R132C
FLT3_D835Y
IDH1_R132H
U2AF1_S34F
KRAS_G12D
NRAS_Q61K
KRAS_G12V

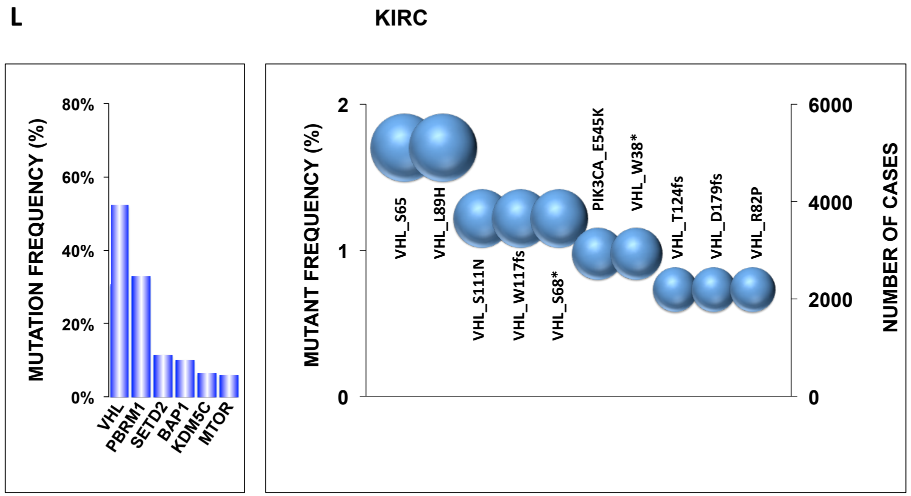
L
KIRC
MUTATION FREQUENCY (%)
80%
60%
40%
20%
0%
VHL
PBRM1
SETD2
BAP1
KDM5C
MTOR
MUTANT FREQUENCY (%)
2
1
0
NUMBER OF CASES
6000
4000
2000
0
VHL_S65
VHL_L89H
VHL_S111N
VHL_W117fs
VHL_S68*
PIK3CA_E545K
VHL_W38*
VHL_T124fs
VHL_D179fs
VHL_R82P
